# *Notes from the Field:* Hepatitis B Virus Transmission Associated with Assisted Blood Glucose Monitoring in a Skilled Nursing Facility — North Carolina, 2024

**DOI:** 10.15585/mmwr.mm7429a2

**Published:** 2025-08-07

**Authors:** Joshua R. Moore, Taylor Breeyear, Camden D. Gowler, Richard Moore, Sydney Will, Dianne Brewer, Taylor Swankie, Sara Beaver, Ashley Vaughn

**Affiliations:** ^1^Communicable Disease Branch, North Carolina Division of Public Health; ^2^Epidemic Intelligence Service, CDC; ^3^Surry County Health Department, Dobson, North Carolina.

**Summary**

**What is already known about this topic?**

During 2008–2019, 15 hepatitis B outbreaks associated with assisted blood glucose monitoring were reported in long-term care facilities. CDC recommends assigning blood glucose monitors (glucometers) to one person.

**What is added by this report?**

Hepatitis B virus (HBV) was transmitted between two residents of a skilled nursing facility who were receiving assisted blood glucose monitoring with shared glucometers. No other potential infection control risks were identified.

**What are the implications for public health practice?**

Sharing glucometers presents a risk for HBV transmission that can be reduced by routine HBV vaccination of persons with diabetes and dedicating individual glucometers to a single resident.

During 2008–2019, 15 reported hepatitis B outbreaks in long-term care facilities were associated with assisted monitoring of blood glucose (AMBG) (*1*), a practice in which a person receives help from another person, such as a health care provider or other caregiver, to measure their blood glucose levels. CDC recommends assigning blood glucose monitors (glucometers) to one person (*2*). The North Carolina Division of Public Health (NCDPH) has messaging consistent with CDC recommendations.* In May 2024, NCDPH was notified by the Surry County Health Department that a hospitalized patient aged 69 years had received a diagnosis of acute hepatitis B virus (HBV) infection immediately after being transferred from a skilled nursing facility. The patient, who had diabetes, had been a resident at the nursing facility for 10 months. Diabetes management included AMBG.

The patient had no documented history of hepatitis B or hepatitis B vaccination and was hospitalized for an unrelated condition. However, viral hepatitis testing was ordered during admission because of symptoms consistent with acute hepatitis, including jaundice, nausea, and fatigue. Testing indicated an elevated liver transaminase level and positive results for hepatitis B surface antigen (HBsAg) and immunoglobulin M antibody to hepatitis B core antigen (IgM anti-HBc). The positive HBsAg and IgM anti-HBc results, in addition to a positive HBV DNA result, confirmed the diagnosis of acute hepatitis B. The North Carolina and Surry County health departments consulted with CDC to investigate potential HBV exposures, assess infection control practices at the skilled nursing facility, and determine the need for notifying and testing other residents at the facility. This activity was reviewed by CDC, deemed not research, and was conducted consistent with applicable federal law and CDC policy.^†^

## Investigation and Outcomes

An infection control assessment revealed that glucometers at the skilled nursing facility were shared among several residents and observed to be disinfected per manufacturer instructions between uses (*2*). No other gaps in infection control practices (e.g., nonadherence to standard precautions and breaches in injection safety) were observed during either of the two infection control assessment site visits.^§^

North Carolina law requires that HBV infections be reported to NCDPH. To identify residents of the skilled nursing facility who had a previously reported HBV infection, NCDPH matched residents to HBV infection events in the North Carolina Electronic Disease Surveillance System (NC EDSS). No residents of the skilled nursing facility with a previously reported HBV infection were identified in NC EDSS. In mid-June, all residents of the skilled nursing facility were tested for HBV infection to identify residents with a chronic HBV infection and who might have been the source of transmission for the patient with acute HBV infection. Testing received by residents included HBsAg, IgM anti-HBc, total antibody to hepatitis B core antigen, and hepatitis B surface antibody. Residents who received a positive HBsAg result also received testing for HBV DNA. Hepatitis B testing identified one facility resident with a previously diagnosed and unreported chronic HBV infection who had lived at the skilled nursing facility since early 2022 and lived in a room close to that of the resident with acute HBV infection. The resident with chronic HBV infection received AMBG from November 2023 to February 2024, an interval that overlapped with the estimated incubation period for the patient with acute HBV infection (9–21 weeks before jaundice onset).^¶^ AMBG records during March–April 2024 indicated that, during this period, both the resident with chronic HBV infection and the resident with acute HBV infection received daily AMBG from one of two devices located on a single medical cart, and the newly infected resident repeatedly underwent AMBG <1 minute following the resident with chronic HBV infection, an interval that might not have allowed for adequate disinfection. The facility reported that no changes in AMBG policies or protocols occurred in the months preceding the March–April review period. Whole genome sequencing analysis demonstrated that the HBV strains infecting both residents were genetically identical.

## Preliminary Conclusions and Actions

This report presents epidemiologic evidence that HBV infection in a skilled nursing facility was transmitted from a resident with chronic HBV infection to another resident through two shared glucometers. Even when disinfection protocols are followed, sharing equipment that is in contact with blood, such as glucometers, can present a risk for HBV transmission. Dedicating glucometers to a single resident and providing routine hepatitis B vaccination to residents with diabetes would reduce the risk for hepatitis B outbreaks in these settings.

In September 2024, NCDPH contacted all previously discharged residents whose time at the facility overlapped with that of the patient with chronic HBV infection. Notification letters sent by certified mail described the potential risk for HBV infection and recommended bloodborne pathogen testing. NC EDSS registry matches were conducted at 6 months and will be repeated at 12 months post-notification to identify any possible additional cases. As of March 2025, no additional cases had been identified.

Guidance from the Advisory Committee on Immunization Practices (ACIP) recommends universal hepatitis B vaccination for persons aged <60 years (*3*). Based on shared clinical decision making, ACIP also recommends hepatitis B vaccination for adults aged ≥60 years at higher risk for hepatitis B, such as those with diabetes (*3*). Clinicians considering providing HBV vaccination to adults aged ≥60 years with diabetes should consider the risk posed by AMBG in skilled nursing facilities (*4*). Hepatitis B vaccination coverage remains low among persons aged ≥60 years with diabetes (*5*). The findings of this investigation highlight AMBG as a risk factor for HBV transmission and provide evidence that skilled nursing facilities might be an appropriate setting to offer hepatitis B vaccination.

## Footnotes

* NC DHHS | Injection Safety

† 45 C.F.R. part 46, 21 C.F.R. part 56; 42 U.S.C. Sect. 241(d); 5 U.S.C. Sect. 552a; 44 U.S.C. Sect. 3501 et seq.

§
Infection Control Assessment and Response (ICAR) Tool for General Infection Prevention and Control (IPC) Across Settings | HAIs | CDC

¶ The incubation period was estimated based on CDC guidance. Viral Hepatitis: Health Care-Associated Outbreak Investigation Toolkit | CDC
